# 2483. Rise in inpatient *Candida auris* cases at a tertiary hospital in Washington, DC during the COVID-19 pandemic

**DOI:** 10.1093/ofid/ofad500.2101

**Published:** 2023-11-27

**Authors:** Daniel Seeger, Matthew Spence, Marc O Siegel, Tara Palmore, Gizachew Demessie, Irvin Ibarra Flores, Jennifer Dien Bard, Margie Ann Morgan, Deisy Contreras, Jose Lucar, Rebecca Yee

**Affiliations:** George Washington University School of Medicine and Health Sciences, Washington, District of Columbia; George Washington University Hospital, Washington, District of Columbia; George Washington University School of Medicine and Health Sciences, Washington, District of Columbia; George Washington School of Medicine & Health Sciences, DC; George Washington University Hospital, Washington, District of Columbia; Children’s Hospital Los Angeles, Los Angeles, California; Children’s Hospital Los Angeles, Los Angeles, California; Cedars-Sinai Medical Center, Los Angeles, California; Cedars-Sinai Medical Center, Los Angeles, California; The George Washington University, Washington, DC; George Washington University School of Medicine and Health Sciences, Washington, District of Columbia

## Abstract

**Background:**

*Candida auris* is deemed an urgent threat because it spreads easily in healthcare facilities, can cause severe infections with high mortality rates, and is resistant to antifungal agents. Since the onset of the COVID-19 pandemic, we detected a rise in inpatient *C. auris* cases. We describe a series of *C. auris* cases at a tertiary hospital in Washington, DC.

**Figure:**
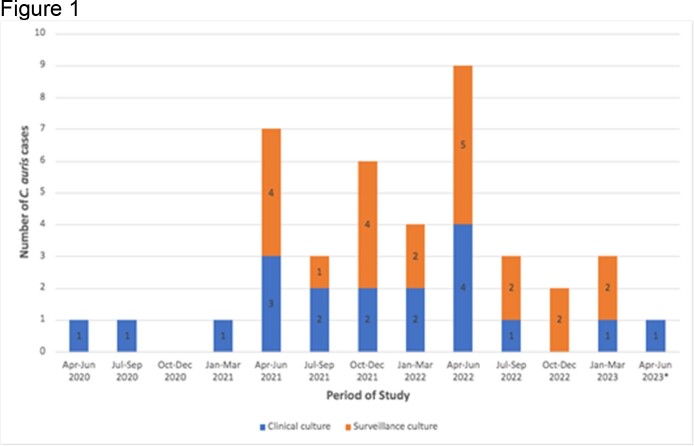

Cases of Candida auris by date of initial sample collection, GW Hospital, Washington, DC, March 2020 - April 2023.

**Methods:**

We reviewed charts of inpatients with *C. auris* between 3/2020-4/2023. Patients transferred from a single long-term care acute hospital (LTACH) with high *C. auris* prevalence were placed in empiric isolation on admission and screened for *C. auris* colonization via axilla and groin surveillance swabs. *C. auris* was identified by MALDI-TOF MS and/or Biofire BCID Panel v2 and strain typing was performed by Fourier-Transform Infrared Spectroscopy. Antifungal susceptibility testing (AST) was performed via broth microdilution and interpreted using tentative breakpoints from CDC.

**Results:**

We identified 41 cases of *C. auris* (median age 62, 66% males). 29 patients (71%) were transferred from the LTACH. 29 (71%) patients had either a tracheostomy or gastrostomy tube. 22 (54%) were identified through surveillance culture (21 from the LTACH) and 19 (46%) via clinical culture (blood=9, urine=7, wound=2, respiratory=1). 2/32 (6%) patients with *C. auris* detected initially via non-blood specimens later developed *C. auris* candidemia. 8/11 (73%) cases of candidemia were CLABSIs. Overall, 8/41 (20%) patients either died or were placed on comfort care during hospitalization. Among 23 isolates available for AST, 22 (96%) were resistant to fluconazole, 15 (65%) to amphotericin B, and 1 (4%) to anidulafungin. Preliminary strain typing suggested grouping of isolates into 2 main clusters denoting distribution into 2 distinct clades; further characterization is undergoing.

**Conclusion:**

The COVID-19 pandemic placed healthcare systems under unprecedented strain which has accelerated the spread of antimicrobial resistant pathogens. The finding that 1 of 5 patients with *C. auris* infection or colonization died/were placed on comfort care underscores the poor prognosis associated with *C. auris*. To combat the spread of *C. auris*, further development of antifungals, rapid diagnostics, and effective infection prevention strategies are needed.

**Disclosures:**

**Tara Palmore, MD**, Abbvie: Grant/Research Support|Gilead: Grant/Research Support|Rigel: Grant/Research Support **Jennifer Dien Bard, PhD**, Abbott Molecular: Grant/Research Support|BioMerieux: Advisor/Consultant|BioMerieux: Grant/Research Support|BioMerieux: Honoraria|Genetic Signature: Advisor/Consultant|Genetic Signature: Grant/Research Support|Luminex: Grant/Research Support|Salve: Stocks/Bonds|Thermo Fisher: Honoraria

